# Confocal Synaptology: Synaptic Rearrangements in Neurodegenerative Disorders and upon Nervous System Injury

**DOI:** 10.3389/fnana.2018.00011

**Published:** 2018-02-15

**Authors:** Maja Vulovic, Nevena Divac, Igor Jakovcevski

**Affiliations:** ^1^Department of Anatomy, Faculty of Medical Sciences, University of Kragujevac, Kragujevac, Serbia; ^2^Department of Pharmacology, Clinical Pharmacology and Toxicology, Faculty of Medicine, University of Belgrade, Belgrade, Serbia; ^3^Institute for Molecular and Behavioral Neuroscience, University Hospital Cologne, Center for Molecular Medicine Cologne, Cologne, Germany; ^4^Experimental Neurophysiology, German Center for Neurodegenerative Diseases, Bonn, Germany

**Keywords:** Alzheimer’s disease, cholinergic synapses, femoral nerve, hippocampus, spinal cord injury, vesicular glutamate transporters, vesicular inhibitory transmitter transporters

## Abstract

The nervous system is a notable exception to the rule that the cell is the structural and functional unit of tissue systems and organs. The functional unit of the nervous system is the synapse, the contact between two nerve cells. As such, synapses are the foci of investigations of nervous system organization and function, as well as a potential readout for the progression of various disorders of the nervous system. In the past decade the development of antibodies specific to presynaptic terminals has enabled us to assess, at the optical, laser scanning microscopy level, these subcellular structures, and has provided a simple method for the quantification of various synapses. Indeed, excitatory (glutamatergic) and inhibitory synapses can be visualized using antibodies against the respective vesicular transporters, and choline-acetyl transferase (ChAT) immunoreactivity identifies cholinergic synapses throughout the central nervous system. Here we review the results of several studies in which these methods were used to estimate synaptic numbers as the structural equivalent of functional outcome measures in spinal cord and femoral nerve injuries, as well as in genetic mouse models of neurodegeneration, including Alzheimer’s disease (AD). The results implicate disease- and brain region-specific changes in specific types of synapses, which correlate well with the degree of functional deficit caused by the disease process. Additionally, results are reproducible between various studies and experimental paradigms, supporting the reliability of the method. To conclude, this quantitative approach enables fast and reliable estimation of the degree of the progression of neurodegenerative changes and can be used as a parameter of recovery in experimental models.

Cells are the basic structural, functional and biological units of tissues and organs. The nervous system, however, has one peculiarity—rather than a single cell, for the proper functioning of the nervous system a connection between two cells, the synapse, is essential. According to the neuron doctrine, chemical synapses are basic building blocks of neural circuitry (Bullock et al., 2005). Typical synapses include a presynaptic axonal terminal, synaptic cleft and a postsynaptic, most commonly dendritic, part of the synapse. The action potential travels through the axon and at its terminal causes voltage-gated calcium channels to open. An influx of calcium causes synaptic vesicles to merge with the membrane and release neurotransmitter, which binds to its receptor—a ligand gated ion channel—and causes changes in the postsynaptic potential (Südhof and Malenka, 2008). Excitatory and inhibitory postsynaptic potentials are integrated, eventually reaching the threshold, which leads to firing of another action potential. This is a simplified, but for our purpose sufficient description of how the nervous system works. Notably, the synapse plays a central role in the transmission of information from one neuron to another (Bullock et al., 2005).

As the size of a single synapse is well below 1 μm, for the most of the previous century research on synapses was based on electron microscopy. Using electron microscopy, the two classical types of synapses were described—an asymmetric, glutamatergic, excitatory synapse where the postsynaptic density appears thicker than the presynaptic one and the symmetric, inhibitory synapse (Peters, 2002). At the turn of the century, several antibodies for different presynaptic markers were developed, which could specifically identify various types of synapses (Takamori et al., 2000; Fujiyama et al., 2001). Together with the improvement of the resolution of confocal laser scanning microscopes, these markers, used for indirect immunofluorescence staining, became instrumental in gaining insight into the functional significance of synaptic remodeling in various nervous system disease models. Here we aim to present several examples where this simple technique was used in conjunction with functional assessments, to correlate synaptic and functional outcome, introducing confocal synaptology as a functionally relevant readout for animal models of neurological disorders. As our examples are focused on the labeling with antibodies against presynaptic markers, we will only briefly touch upon other labeling possibilities, as well as other technical issues of light microscopy in this context.

## Technical Clarifications

**Figure 1:**
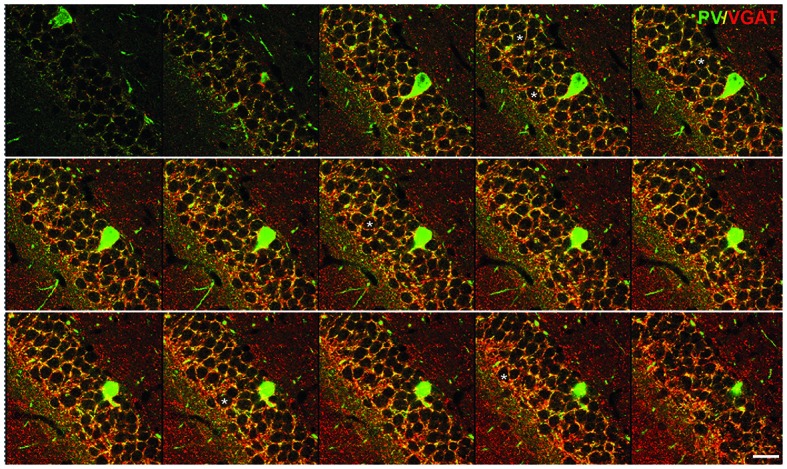
Inhibitory synapses in the dentate gyrus (DG) of the hippocampus. Immunofluorescent co-staining for VGAT (red) and parvalbumin (PV; green) in the wild-type mouse hippocampus. A serial stack of images of 1 μm thickness through the DG obtained on a confocal microscope. Asterisks label several examples of the cells used for the analysis. Scale bar: 20 μm. This figure contains images from the study originally published in Schmalbach et al. (2015).

**Figure 2:**
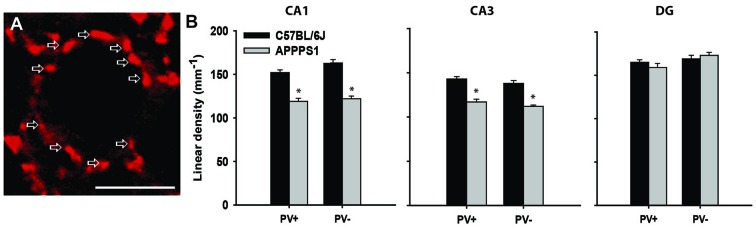
Inhibitory synapses in the hippocampus of Alzheimer’s disease (AD) model mice. **(A)** Immunofluorescent staining for VGAT (red) of a single pyramidal neuron in the CA1 of a wild-type mouse. Arrows point to single terminals. **(B)** Linear density (number of terminals per unit length) of inhibitory (VGAT+) synapses, further subdivided into PV+ and PV− terminals in the wild-type (C57BL/6J) and AD model (APPPS1) hippocampi. Data are presented as mean + standard error of the mean, asterisks indicate *p* < 0.05, *t*-test; *n* = 5 mice/group. Scale bar: 10 μm. This figure contains images and data from the study originally published in Djogo et al. (2013).

**Figure 3:**
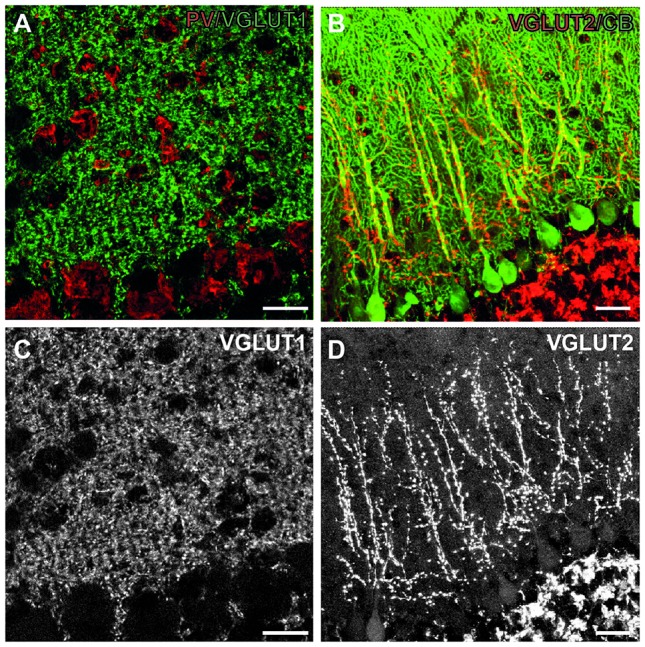
Excitatory synapses in the cerebellum. **(A)** Immunofluorescent co-staining for VGLUT1 (green) and PV (red) in the wild-type mouse cerebellum. Shown are Purkinje cell layer (bottom part of both images) and the molecular layer (upper part of images), where these synapses are located. **(B)** Immunofluorescent co-staining for VGLUT2 (red) and calbindin (green) in the wild-type mouse cerebellum. **(C,D)** Single images from A **(C)**, B **(D)** showing only VGLUT1 and VGLUT2 staining, respectively. Note diffuse staining pattern in the molecular layer with VGLUT1, leaving only putative interneurons unstained. On the contrary, VGLUT2 staining is more discrete, and confined to the main branches of Purkinje cell dendrites. Scale bars: 20 μm. This figure contains images from the study originally published in Jakovcevski et al. (2009).

**Figure 4:**
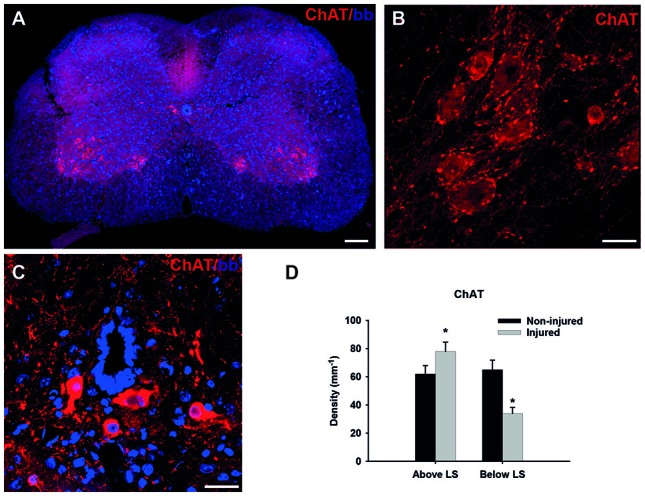
Cholinergic synapses in the spinal cord. **(A)** Immunofluorescent staining for choline-acetyl transferase (ChAT; red) of the non-injured wild-type mouse spinal cord transverse section in the lumbar region. **(B)** Higher magnification of motoneurons in the ventral horn, red dots around cell bodies represent cholinergic C-terminals. **(C)** Cholinergic interneurons around central canal, a source of C-terminals on motoneurons. **(D)** Linear density (number of terminals per unit length) of cholinergic (ChAT+) synapses in wild-type (C57BL/6J) of non-injured and injured mice above and below the lesion site (LS). Data are presented as mean + standard error of the mean, asterisks indicate *p* < 0.05, *t*-test; *n* = 6 mice/group. Scale bars: 100 μm **(A)**, 20 μm **(B,C)**. This figure contains data and images from the study originally published in Jakovcevski et al. (2007, 2013a).

Antibodies will visualize synapses best when they have higher affinity for terminals rather than axons. To that end, antibodies for vesicular transporters for inhibitory (VGAT) and excitatory (VGLUT) transmitters are ideal, as they specifically label presynaptic terminals (Figures 1–3). As the inhibitory (mainly gamma-aminobutyric acid—GABA—positive) synapses target mostly cell bodies and proximal dendrites of neurons, we count the number of VGAT-expressing puncta surrounding the neuron cell bodies and normalize it to the cell perimeter (Figure 1; original data published in Schmalbach et al., 2015). This method was used on the pyramidal cells of the cortical layer 5 (Irintchev et al., 2005), hippocampal principal neurons (pyramidal neurons in the Amon’s horn—CA, and granule cells of the dentate gyrus—DG; e.g., Nikonenko et al., 2006; Radonjić et al., 2013), cerebellum Purkinje neurons (Jakovcevski et al., 2009), and spinal cord motoneurons (Apostolova et al., 2006). To standardize the perimeter length stacks of images of 0.5 or 1 μm thickness are obtained on a confocal microscope using 63× or 100× oil immersion objectives and at a high digital resolution (e.g., 1024 × 1024 or 2048 × 2048 pixels would suffice). One image per cell at the level of the largest cell body cross-sectional area is used for measurement (Figure 1). The number of individually discernible structures is counted and then normalized to the perimeter of the cell measured (Figure 2A). A similar method is applied for the estimation of the cholinergic, choline-acetyl transferase (ChAT)-positive, terminals around spinal cord motoneuron cell bodies (Davidoff and Irintchev, 1986; Apostolova et al., 2006). Although ChAT antibody stains motoneuron cell bodies and axons, immunoreactivity is strongest in the presynaptic terminals (Figure 4). Thus, the number of cholinergic synapses can also be normalized to the cell perimeter (length). Alternatively, the antibody for vesicular acetylcholine transporter can be used, having the similar staining pattern (Schäfer et al., 1998).

With regard to the far more numerous glutamatergic, excitatory synaptic terminals the situation is not as simple. Whereas both main inhibitory transmitters, GABA and glycin are packed in VGAT-expressing vesicles, there are at least three types of vesicular transporters for glutamate, VGLUT1-3 (Liguz-Lecznar and Skangiel-Kramska, 2007). These three types are mostly complementary, with little overlap, VGLUT1 being the main transporter in the cerebral cortex and hippocampus, while VGLUT2 is expressed in the granular layer of the DG, thalamus, hypothalamus and brainstem (Fujiyama et al., 2001; Fremeau et al., 2004). In the cerebellum, parallel fibers express VGLUT1, whereas climbing fibers express VGLUT2 (Hisano et al., 2002). VGLUT3 distribution is restricted to particular populations of neurons, furthermore it is expressed in a subpopulation of inhibitory synapses in the hippocampus and neocortex, as well as cholinergic and serotonergic neurons. This lead to an idea that glutamate could be co-released with another transmitter and modulating its effects (Liguz-Lecznar and Skangiel-Kramska, 2007). Therefore, VGLUT3 cannot be used as a reliable marker for glutamatergic transmission. To estimate glutamatergic transmission using VGLUT1 or VGLUT2 digital images obtained on a confocal microscope are processed with ImageJ software (NIH, Bethesda, MD, USA). The gray value is adjusted using threshold to convert the images to gray scale for optimal color intensity with ImageJ. Criteria for identification of synaptic terminals may vary depending on the brain region with respect to object size and circularity. Density is calculated as the number of VGLUT1^+^ synaptic terminals per unit area. One should emphasize that the expression “linear density” (number of terminals per unit length) and “density” (number of terminals per unit area) should not be confused with the “synaptic density” (as in pre- and post-synaptic densities) commonly used in electron microscopy.

In addition to the possibility to combine the presynaptic terminal markers with cell-type specific markers, e.g., VGAT with parvalbumin (PV), to focus on a specific type of synaptic input (see below), it is also possible to use both presynaptic and postsynaptic markers, to ensure the more precise definition of the synapse. This analysis is more laborious and demanding, but are more likely to quantify true synapses, especially when combined with super-resolution microscopy and 3D reconstruction (Janz et al., 2017). Postsynaptic markers such as post-synaptic density protein 95 (PSD-95), localized exclusively to excitatory synapses (Hunt et al., 1996) and gephyrin, localized to inhibitory synapses (Sassoè-Pognetto and Fritschy, 2000) are a good choice to be combined with VGLUT1 and VGAT, respectively. However, the double-labeling is often burdened with unequal penetration of the two antibodies, which makes analysis more difficult (Janz et al., 2017). Additionally, about 20% of dendritic spines lack PSD-95, although it is not clear if these spines are functionally significant, which further complicates the interpretation of results (Berry and Nedivi, 2017).

Another way to address the specificity of synaptic labeling would be by using genetic mouse models or viruses encoding fluorescent proteins expressed by pre- or postsynaptic terminals. They have clear advantages over the classical immunostaining, but breeding the animals is time consuming, and viral injections are invasive and expression/transduction is usually incomplete, so their utilization is very much dependent on the experimental settings.

Although the power of electron microscopy is unquestionably higher in revealing the ultrastructure of neural tissue, recent advances of fluorescence light microscopy are directed to overcome the diffraction limit of the visible light (the resolution limit of 200 nm), as the main obstacle to resolve morphological details at the subcellular and even molecular level (Dani and Huang, 2010). Combined with the inherent advantages of fluorescence microscopy, including possibility to visualize protein colocalization, cell-specific protein marker expression, and various forms of genetic or dye-based labeling, even the possibility to work with cell or tissue cultures, the new super-resolution techniques which achieve resolutions up to 20 nm are opening new possibilities for Huang et al. (2008).

## The Role of the Close Homolog of L1 in the Development and Aging of Inhibitory Synapses in the Hippocampus

One of the first studies to show that estimation of the synaptic coverage by confocal microscopy corresponds well with the “gold standard” electron microscopic method was the investigation of the hippocampus in juvenile close homolog of L1 (CHL1)—deficient mice (Nikonenko et al., 2006). Similar to L1, its homolog CHL1 is an immunoglobulin superfamily adhesion molecule implicated in axonal growth and pathfinding, neuronal migration and synapse formation (Demyanenko et al., 2004; Wright et al., 2007; Katic et al., 2014). Among others, it has been associated with schizophrenia and autism spectrum disorders in humans (Angeloni et al., 1999; Chen et al., 2005). Mice constitutively lacking CHL1 (CHL1−/−) show behavioral deficits congruent with psychiatric disorders (Irintchev et al., 2004; Kolata et al., 2008). A role of CHL1 in the establishment of the inhibitory circuitry has been demonstrated in the CA1 field of the hippocampus. Juvenile (1-month-old) CHL1−/− mice develop increased numbers of PV-expressing (PV+) interneurons in the CA1, which account for the most of the fast-spiking interneurons in this area (Gulyás et al., 1999). In this study the estimation of the symmetric, inhibitory perisomatic synapses by electron microscopy was performed in parallel with the counting of VGAT+ inhibitory synapses abound the cell bodies of CA1 pyramidal neurons. Both methods showed increased numbers of perisomatic synapses in CHL1−/− compared with wild-type animals, the latter being capable of distinguishing between PV+ and PV− terminals, which were both affected to the approximately same degree (Nikonenko et al., 2006). To test for the physiological relevance of this apparent increase in the inhibition, authors measured inhibitory postsynaptic currents (IPSCs) in the presence of a glutamate receptor antagonist. The mean amplitude of IPSCs was increased in CHL1−/− mice compared with wild-type controls. As GABAergic transmission was apparently increased in CHL1−/− mice, further hypothesis was that this abnormality might lead to a reduction of long-term potentiation (LTP), an electrophysiological correlate of memory formation (Bliss and Lomo, 1973; Nicoll, 2017). To verify this hypothesis, authors stimulated axons of CA3 neurons and recorded field excitatory postsynaptic potentials (fEPSPs) in the stratum radiatum of the CA1 region. LTPs evoked by five trains of theta-burst stimulation were significantly reduced in CHL1−/− as compared with wild-type mice. To show that the increased inhibition is the cause of this deficit, the authors blocked the GABA_A_ receptors with picrotoxin, which lead to similar LTPs in both genotypes (Nikonenko et al., 2006). Thus, the increased inhibition causes the reduction of LTP, i.e., functional deficit in juvenile CHL1−/− mice. Interestingly, the follow-up study which analyzed the senescence of CHL1−/− mice, has shown that the number of PV+ interneurons normalizes in adult, 2-month-old mice and at later ages, 6 and 12 months, they are reduced in number compared to wild-type. Congruently, LTP normalizes at 2 months, but later, at 9 months it decreases again, relative to the wild-type values (Schmalbach et al., 2015). This example shows that the alteration in specific synapses can be correlated with functional deficits, and that this correlation can progress during animal lifespan. This may be relevant for the investigation of complex human-specific diseases, such as schizophrenia, as, besides the obvious connection with the loss of PV+ interneurons, some other neurological and immunological features in CHL1−/− mice resemble the pathology seen in schizophrenic patients (Schmalbach et al., 2015).

## Alzheimer’S Disease: The Loss of Hippocampal Inhibitory Synapses

Alzheimer’s disease (AD) is commonly diagnosed disorder in the elderly characterized by degenerative changes in neurons, amyloid plaques, neurofibrillary tangles, and progressive diminishing of working memory and intellectual capacities (Querfurth and LaFerla, 2010). The most commonly used mouse model of this disorder is generated by overexpressing human mutated amyloid precursor protein, so-called “Swedish mutation” (Radde et al., 2006). These mice display cerebral amyloidosis, gliosis and later in life a working memory deficit. As there is no therapy for cerebral amyloidosis, and for AD, we tried an experimental treatment with gene transfer, as a proof-of concept study. To that end we used neural adhesion molecule L1, shown in various experimental neural disease paradigms to be neuroprotective and gliosis limiting agent (Chen et al., 2007; Cui et al., 2010; Xu et al., 2011; Lee et al., 2012), and overexpressed it in the hippocampus of the transgenic mouse model for AD (Djogo et al., 2013). Using adeno-associated virus serotype 5, we achieved a robust L1 expression in neurons and in astrocytes in the hippocampus. The beneficial effects seen in L1 treated group of mice included a decrease in amyloid plaque load and in astrogliosis, and, from the functional point most significantly, ameliorated loss of inhibitory synapses on the hippocampal pyramidal neurons. At 8 months of age, when the number of neurons is still normal in the hippocampus of these mice (Radde et al., 2006), the perisomatic VGAT-positive inhibitory synapses around principal neurons were reduced in the CA1 and CA3 fields, but not in the DG (Figure 2B). This was true for both PV-positive and PV-negative inhibitory synapses, suggesting decreased overall local circuitry inhibition (Figure 2). L1 transduction reduced synaptic loss in affected areas, although it could not completely protect the synapses (Djogo et al., 2013). This example shows how synaptic loss can be an early predictor of disease development, and a functionally relevant structural parameter to estimate treatment efficacy, as synaptic loss is a major correlate of cognitive impairment in human AD patients (Terry et al., 1991; Arendt, 2009).

## Cerebellum Development

The cerebellar cortex is particularly interesting, as it has one of the best described circuitries. Purkinje cells are its principal neurons contacted by the afferent climbing fibers originating from the brainstem. Granule cells constitute the major population of excitatory interneurons which project their axons, the parallel fibers, to the Purkinje cell dendrites. The inhibitory stellate and basket interneurons also target their axons onto the dendrites and cell bodies of Purkinje cells. This circuitry has an additional appeal, as the excitatory input is particularly well defined. The integrated sensory information comes from the inferior olives as climbing fibers (VGLUT2-positive), which directly innervate Purkinje cell dendrites. Various other brainstem areas project mossy fibers (VGLUT1-expressing), which innervate granule cells, which in turn project parallel fibers (also VGLUT1-positive) through molecular layer onto Purkinje cell dendrites. The sole output of the cerebellar cortex are Purkinje neuron axons, which are inhibitory, express VGAT and calbindin and synapse on the deep cerebellar nuclei. Interestingly, whereas parallel fibers innervate many (approximately thousand) Purkinje cells, each climbing fiber innervates a single Purkinje neuron (Cajal, 1911; Eccles et al., 1966).

In CHL1−/− mice the cerebellar cortex develops lower numbers of Purkinje and granule neurons, with normal numbers of interneurons, which indicates an increased inhibition/excitation ratio. We, therefore, studied the inhibitory, VGAT-positive terminals on Purkinje cell bodies and dendrites, and discovered that their density was similar to the wild-type. The density of VGLUT1-positive (parallel fibers) terminals in the molecular layer was normal as well. The only difference was in the decreased number of VGLUT2-positive climbing fiber terminals, congruent with the lower number of Purkinje neurons, as the single climbing fiber innervates single Purkinje cell (Figure 3). Our next hypothesis was that branching of Purkinje cell dendrites was compensatory increased in CHL1−/− mice, which indeed was the case (Jakovcevski et al., 2009). It is tempting to hypothesize that developmental compensation at the level of Purkinje cell dendrites accounts for the lack of the functional cerebellar phenotype in CHL1−/− mice. This exemplifies how the presynaptic changes could be compensated through the changes on the postsynaptic side so that the overall function is not disturbed. It also emphasizes the importance of studying both the presynaptic terminals and dendritic spines to fully understand the synapses and their functional significance.

## Spinal Cord Injury

Spinal cord injury is a severe neurological disorder which affects mostly young people and greatly reduces both life expectancy and quality (Ahuja et al., 2017). As there are no reliable therapies, regeneration after injury is a major target of research in neurobiology, ever since Cajal observed and postulated that central nervous system axons do not regenerate (Cajal, 1928). The spinal cord has an extremely intricate circuitry, details of which are still largely unknown, with many types of interneurons, some of which are probably as of today unrecognized (Bikoff et al., 2016). One well-described part of this circuitry are the cholinergic V0 interneurons which form large C-type synapses on alpha motoneurons in spinal cord segments below them (Zagoraiou et al., 2009). Those synapses are currently thought of as the modulators of motoneuron firing (Miles et al., 2007). Importantly, they are clearly visualized with ChAT antibodies which stain motoneuron cell bodies as well, but they stain the terminals particularly strong (Figures 4A–C). These cholinergic synapses are reduced in number after spinal cord injury (Apostolova et al., 2006). Interestingly, this reduction is true for the cholinergic synapses on motoneurons below the lesion site (LS), whereas above the LS the number of these synapses after injury is slightly increased (Figure 4D; Jakovcevski et al., 2013a). This underlines the complexity of synaptic re-arrangements after spinal cord injury.

It has been shown that upon spinal cord injury CHL1−/− mice recover locomotor function better than their wild-type littermates, due to the homophilic CHL1-CHL1 signaling between astrocytes and neurons, which interferes with axonal regeneration (Jakovcevski et al., 2007). In this mouse model we have shown that the reduction of cholinergic terminals 6 weeks after injury positively correlates with the severity of motor deficit on an individual animal basis. This was done using linear regression and comparing the density of cholinergic synapses with the “gold standard” BMS score (Basso et al., 2006), as well as the single-frame motion analysis locomotor scoring (Apostolova et al., 2006). Several other studies in the mouse model of spinal cord injury have consistently reported that the higher number of cholinergic synapses around motoneurons corresponds to the improved locomotor outcome (Cui et al., 2011; Xu et al., 2011; Lee et al., 2012; Wu et al., 2012; Papastefanaki et al., 2015; Rost et al., 2016; Saini et al., 2016). We additionally quantified VGAT-positive inhibitory synapses around motoneuron cell bodies, as well as glutamatergic synapses, but the changes among those were not consistent and are difficult to explain in terms of possible connections with functional recovery (Jakovcevski et al., 2007).

A further breakthrough was achieved while studying another adhesion molecule, extracellular matrix glycoprotein tenascin-C. Tenascin-C, as a part of the extracellular matrix, plays important roles in neural development and plasticity, but also wound healing (Jakovcevski et al., 2013b). Tenascin-C−/− mice recover worse than their wild-type littermates after spinal cord injury, indicating a positive role that tenascin plays in regeneration (Chen et al., 2010). In these animals we measured the Hoffman (H) reflex, as an electromyographic correlate of a monosynaptic tendon reflex, and a commonly taken measure of motoneuronal excitability in both humans and experimental animals (Knikou, 2008), aimed to clarify the relationships between functional recovery, spinal reflex properties and spasticity after spinal cord injury (Lee et al., 2009). Upon the electrical stimulation of the sciatic nerve, plantar muscle response is measured. The first response comes immediately, so-called M-wave, the product of direct stimulation of muscle though anterograde conduction of the spinal nerve. The stimulus is, however, also retrogradely conducted through Ia afferent to motoneuron and then back to the muscle. This, so-called “H” wave is a measure of motoneuron reflex integration. In the chronic phase of spinal cord injury, the H-reflex is facilitated upon single and repetitive low-frequency stimulation (Thompson et al., 1992). As in the previous literature, we confirmed that, compared to the intact spinal cords, H-reflex is facilitated early (7 days) after injury, and remains high throughout the follow-up of 12 weeks (Lee et al., 2009). Unexpectedly, however, the reflex moves closer to normal, lower values in TNC−/− mice, which recover worse, and remains higher in CHL1−/− mice, which recover better after injury (Lee et al., 2009; Chen et al., 2010). The same trend—higher H-reflex values compared to wild-type injured mice—was seen in tenascin-R−/− mice, another mouse model with facilitated functional recovery after injury (Lee et al., 2009). These data indicated that spasticity after injury could play an important role in functional recovery during the subacute phase. We proceeded to assess glutamatergic, excitatory synapses in wild-type and tenascin-C−/− mice, to search for the possible structural correlates of the observed spasticity (Chen et al., 2010). We analyzed glutamatergic input in three spinal cord areas in which VGLUT1-positive terminals were prominent: the Clarke’s column, which receives proprioceptive information from dorsal roots, an adjacent part of lamina VII, an area in the spinal cord containing last-order interneurons, i.e., innervating motoneurons, lamina IX, where proprio- and mechanoreceptors form contacts predominantly on motoneuron dendrites. Analysis of wild-type and tenascin-C−/− mice 12 weeks after injury revealed a significant, compared with non-injured mice, decline in the terminal densities in the Clarke’s column and lamina VII, and a strong increase in lamina IX in both genotypes. The conspicuously lower numbers of VGLUT1-positive terminals in lamina VII of tenascin-C−/− mice could lead to speculation that this is the structural correlate of lower H-reflex values in these mice (Chen et al., 2010). The same method of analysis was applied to assess the role of polysialic acid (PSA) mimicking peptide in facilitating recovery upon spinal cord injury, and mice treated with PSA-mimic had higher numbers of VGLUT1-positive terminals in both laminae VII and IX compared with vehicle-treated animals (Mehanna et al., 2010). This adds weight to the evidence that higher glutamatergic transmission in laminae VII and IX of injured spinal cords indicates better functional outcome.

## Femoral Nerve Injury

Although the peripheral nervous system recovers from injury to a considerably higher degree than the central nervous system, injury to peripheral nerves still presents an important clinical problem, causing a significant degree of disability in patients (Navarro et al., 2007; Irintchev, 2011). The femoral nerve is a mixed nerve which divides into two terminal branches, a muscle branch to the quadriceps muscle and a cutaneous branch, the saphenous nerve. Injury immediately proximal to the bifurcation of the two branches affects a single muscle, the quadriceps. After surgical repair, the quadriceps motor axons have an equal opportunity to regrow into the original pathway, the quadriceps branch, or choose the wrong pathway, the purely sensory saphenous nerve (Akyüz et al., 2013). This situation allows analyses of cellular and molecular mechanisms involved in specificity of motor reinnervation (Brushart, 1993; Robinson and Madison, 2005). In addition, the model is interesting because femoral nerve injury causes disability of a single muscle, the quadriceps, which functions as a knee extensor, thus allowing a simple approach for locomotor analysis (Irintchev, 2011). The factors limiting recovery after nerve injury become even more complicated if we consider the complexity of central nervous system reorganization after peripheral nerve injury (Navarro et al., 2007). To clarify some of this complexity, analysis of the synaptic remodeling after injury is essential. After nerve injury motoneurons are acutely deafferentiated and upon reinnervation of their target muscles they regain their synaptic afferents to varying degrees, as demonstrated by electron (Brännström and Kellerth, 1999) and light microscopy (Hellstrom et al., 2003).

Both cholinergic (ChAT-positive) and inhibitory (VGAT-positive) terminals around motoneuron cell bodies were studied by light microscopy in various mouse models of femoral nerve injury and surgical repair (Simova et al., 2006; Guseva et al., 2009, 2011, 2014; Mehanna et al., 2014). Here, the linear density of perisomatic terminals immunoreactive for ChAT or VGAT was estimated around motoneurons retrogradely labeled at 3 months after injury. Retrograde labeling is important in this case, to take into account only injured motoneurons (i.e., those that give rise to the femoral nerve), and to avoid taking neurons which upon repair falsely projected through the sensory branch, and thus play no role in motor recovery (Akyüz et al., 2013). Consideration must be given, however, that the retrograde labeling procedure involves a second surgery and nerve resection to apply the dye, which is typically conducted 1 week before sacrifice of the animals. Thus, the results of such analysis take into account data about simultaneous acute and chronic reaction of the nerve to injury. Both cholinergic and inhibitory perisomatic terminals were reduced in numbers 2 months after femoral nerve injury (Mehanna et al., 2014), whereas at 3 months after injury only inhibitory terminals were reduced (Guseva et al., 2009, 2011), suggesting plasticity in the sub-acute and chronic phases of regeneration. To drive solid conclusions, however, about the central synaptic rearrangements after peripheral nerve injury further studies are necessary.

## Conclusions and Outlook

We hope that by presenting several studies which combined functional and anatomical assessment, we add to the evidence that confocal synaptology is a reliable and convenient method to address various questions in neurobiology. Although there is no replacement for electron microscopy, as the standard method for visualization and morphological analysis of synapses, confocal synaptology presents itself as an excellent screening method, to assess the regions of interest and types of synapses to be analyzed by electron microscopy. As with all methods, it is of great importance to understand the limitations of this type of analysis. Although we have shown that in some cases, for particular types of synapses, the quantitative estimation achieved by this technique corresponds well with the functional parameters, the results should always be interpreted cautiously, and when possible attempted to correlate with the functional parameters. It should also be noted that the numerical values, in this case, are rather “synaptic score values” than the actual numbers of synapses, as synapses are precisely quantifiable only by electron microscopy. The advancement of super resolution fluorescence microscopy, however, will likely eventually blur the advantage of electron microscopy for the visualization of synapses (Dani and Huang, 2010). Further studies are necessary to conclude in which cases the results of the light microscopic estimation of synaptic numbers and changes renders functionally relevant results, which will increase our understanding of the intricate circuitries of the central nervous system.

## Author Contributions

MV, ND and IJ were involved in analyzing the literature and writing the manuscript.

## Conflict of Interest Statement

The authors declare that the research was conducted in the absence of any commercial or financial relationships that could be construed as a potential conflict of interest.
